# Listen and You Will See the Person Through the Dementia

**DOI:** 10.17505/jpor.2021.23797

**Published:** 2022-01-13

**Authors:** Rita Estrada

**Affiliations:** Instituto Superior de Ciências Educativas do Douro, Penafiel, Portugal

**Keywords:** dementia, listening, person, basic psychological needs, relatedness, competence, autonomy

## Abstract

Dementia is an ever-increasing health and social problem, with a growing number of people being affected worldwide. As dementia progresses, dependency on others increases, requiring the presence of caregivers. Caregivers tend to focus on the diagnosis itself – dementia – which makes it difficult to see the person in their uniqueness. The person is there, and can be seen by listening, which requires time and communication skills. The voices of older adults living with several types of dementia, collected while working as a psychologist in a nursing home, are presented in the first person to bring forward the person they are. These excerpts of interactions illustrate the basic psychological need of relatedness, which is built through interaction, stories, and touch, and the needs of competence and autonomy. The framework of this paper en-compasses validation therapy, person-centered care, and self-determination theory. Two conclusions emerge: Seeing the person through the dementia enables an adequate psychological assessment and a helpful supportive psychotherapy, and it also makes us acknowledge and help satisfy the three basic psychological needs of relatedness, competence, and autonomy.

Dementia is an ever-increasing health and social problem, as numbers demonstrate: Around 50 million people world-wide have dementia, and an additional 10 million are expected to be affected each year (World Health Organization, n.d.). Dementia or neurocognitive disorder (NCD) consists of a neurodegenerative process characterized by the presence of cognitive deficits that affect one or more cognitive domains, such as orientation (e.g., not being able to locate oneself in space and time), attention (e.g., having difficulties in retaining simple information provided in the moment), memory (e.g., keep telling the same things to the same people), language (e.g., having difficulties in finding words) (American Psychiatric Association [APA], 2013; Spar & La Rue, 2002). Dementia has no cure so far; its course and severity depend on several factors: age, gender, age of onset, type of dementia (e.g., due to Alzheimer’s or Parkinson’s disease, or vascular NCD), comorbidities, past and actual lifestyle (Spar & La Rue, 2002; Kurudamannilb & Hemachandra, 2019). These factors also help to explain the variation in life expectancy, from one year to a couple of decades.

The life expectancy of a person living with dementia is a central issue as it makes us question how one lives with dementia for years, and whether one’s basic psychological needs are satisfied or not. As dementia is characterized by a series of progressive losses, associated with the cognitive deficits, caregivers (health professionals, care staff, family) easily forget there is a person there, not acknowledging the basic psychological needs, and focusing mainly on the basic survival needs. However, the person is there, as are also the basic psychological needs of relatedness, competence, and autonomy. This is seen through listening, as this paper demonstrates, by giving voice to older people living with dementia. All pieces of discourse were collected while working as a psychologist in a Portuguese nursing home. Contact with older adults living with dementia was established on a daily basis in formal settings: neuropsychological assessment, neuropsychological rehabilitation sessions, and along with supportive psychotherapy meetings. Interaction was also established in informal settings, such as mealtimes. The voices’ authors are older adults living with mild and major NCD.and along with supportive psychotherapy meetings. Interaction was also established in informal settings, such as mealtimes. The voices’ authors are older adults living with mild and major NCD.and along with supportive psychotherapy meetings. Interaction was also established in informal settings, such as mealtimes. The voices’ authors are older adults living with mild and major NCD.^1^1Names and identifying details had been changed to protect privacy, following the orientations given by the Ethics Board of the Portuguese Psychologists Association.


This paper begins by exploring the process of listening, which allows seeing the person through the dementia. This process rests on validation therapy principles (e.g., Feil, 1985, 1992) and person-centered care for people living with dementia (e.g., Brooker, 2019; Love & Pinkowitz, 2013). In the second section, the paper then illustrates how older adults living with dementia continue experiencing the basic psychological needs of relatedness, competence, and autonomy (Ryan & Deci, 2000). The paper concludes by stating that seeing the person through the dementia enables an adequate psychological assessment and a helpful supportive psychotherapy, and it also makes us acknowledge and help satisfy the basic psychological needs.

## Listening

A large percentage of nursing homes’ residents have NCD with diverse degrees of severity. Diagnoses are established based on a multidisciplinary evaluation. The psychologist’s role consists in an assessment of the person based on a clinical interview and standardized neuropsychological tests. The objectivity and structure of the evaluation process needed for the establishment of a diagnosis has two short-comings: the limitation of time, making it difficult to listen and relate to the person, and the limitation of focusing too much on the diagnosis itself.

The American psychologist Richard Taylor (2007), who was diagnosed with Alzheimer’s disease, warns against the propensity to put too much emphasis on the diagnosis, and belittle the person and their individuality, which do not vanish when living with dementia. Taylor makes use of a distinction advanced by Buber (1923/1970) between “I-Thou” and “I-It” relations; concepts cited widely and in different fields such as theology, philosophy of education, philosophical psychology, and medical anthropology (Zank & Braiterman, 2020). In “I-Thou” relations there is reciprocity, each part is recognized as an equal, and treated with respect. In “I-It” relations there is a process of reification of the other, who is used as a means to an end. Using these terms, Taylor (2007, p. 149) makes an appeal: “I do know that I continue to need to be recognized as a Thou, to have my personhood recognized. Please understand, I am still here.”

The question that arises is how to go beyond the diagnosis of dementia and be capable of seeing the person. The answer is: Listen. It is when we listen to older adults living with dementia that we see them as persons, unique beings, with their own life stories. When we listen, we begin to interact along an I-Thou relation, characterized by a respectful and sensitive care, which gradually allows an identity, needs, likes and dislikes, and conflicts to emerge (Deutscher Ethikrat, 2013; Feil, 1985, 1992).

It is not a simple and immediate process. As a psychologist in a nursing home, what one first sees are deficits: lack of orientation in time and space, lack of recognition of us, lack of memory. This is also the most common representation of dementia, as portrayed for instance in the 2020 movie *The father* directed by Florian Zeller – a portrait of deficits and losses. Notwithstanding, by listening and interacting, we see the person through the dementia, a practice acknowledged a long time ago by Feil (1985) and Kitwood (1997/2019a). However, it is still far from being recognized and practiced globally, and still “seen as the unreachable ideal” (Brooker, 2019, p. 82).

Mrs. Fernanda was 86 years old and had been diagnosed with major NCD with behavioral disturbance, by DSM-5 criteria (APA, 2013) ^2^2Information about age and severity of the NCD is given when a resident is referred for the first time. All diagnoses by DSM-5 criteria (APA, 2013).. She was in a wheelchair. Mrs. Fernanda regretted her present situation and kept saying “what I was and what I am now”. She felt lonely, she had tactile hallucinations that scared her, and she manifested strong agitation, screaming very loud. One day, she told me “screaming is my whistle to get attention”. Mrs. Fernanda needed to be listened to and to be seen. Offering that to her was the way to calm her down, to give her safety, and quality time.

Mrs. Manuela, 98, diagnosed with major NCD, was in a wheelchair. She longed to be listened to and understood. After six meetings, she said “little by little you are entering in my mind”.

These two excerpts of discourse illustrate a type of interaction with older people living with dementia influenced by the validation method (Feil, 1985, 1992) and by the person-centered care for people living with dementia (Brooker, 2019). The point here is that through interaction deficits can start to lose prominence, and the individual is seen.

Kitwood criticized the biomedical model centered on the disease, proposing a new paradigm with the motto “the person comes first” (Kitwood, 1997/2019a, p. 2). Since then, person-centered care for adults living with dementia has gone through a few definition refinements, and is actually put into practice in some nursing homes. Its guiding principles are the following: To reaffirm the person’s value as a human being, showing that we see a You, not an It, respecting and valuing the person’s dignity and self-determination; to recognize individuality and agency, which presupposes knowing the life story, acknowledging strengths, and respecting values and choices; to attend to psychological needs; to have an empathic attitude towards the experience of living with dementia; to pursue a meaningful life for people living with dementia; to advocate for a holistic care by looking at the person in all dimensions; to interact in a caring and genuine way (Brooker, 2019; Cheston, 2019; De Medeiros & Doyle, 2013; Deutscher Ethikrat, 2013; Downs, 2013; Kirtley & Williamson, 2016; Kogan et al., 2016; Love & Pinkowitz, 2013).

On the other hand, when we see the person through the dementia, it is possible to observe performance in diverse cognitive functions in natural social environment. In this way we obtain information that complements or even modifies data gathered via standardized tests (Sabat & Cagigas, 1997). One of the most impressive examples I experienced happened with Mrs. Maria José, 76, with a major NCD due to Parkinson’s disease, with dysarthria (speaking difficulty), in a wheelchair. We went outside the nursing home for a walk, and she asked me to go to a beauty store. When we got inside, she said in a clear voice: “Good afternoon! Do you have eyeliner?” Actually, the environment had been a stimulus for Mrs. Maria José to react, starting a dialogue, and asking for what she wanted. She was also capable of articulating words in a comprehensible way. Nothing of this had occurred during assessment or neuropsychological rehabilitation sessions.

The whole process of listening involves two fundamental aspects – time and communication skills –, which are present in every interaction, though cognitive impairments of dementia make their presence more salient and necessary (Downs, 2013; Kogan et al., 2016). Communication skills specifically are part of training and education programs in dementia care (e.g., Downs, 2013; Kogan et al., 2016).

### Time

Listening presupposes time; one needs to pause, to stay with the older person living with dementia, and to clearly demonstrate that one is there – it is a therapeutic kind of presence (Kitwood, 2019c).

Older people living with dementia recognize and appreciate the time we give them, and they also notice when we do not have time. Let us listen. Mrs. Manuela, with whom I used to be in a context of supportive psychotherapy, once said: “thanks for this little bit, these little bits”. Mrs. Fernanda, after a visit to the garden, said “it was already good for the day”.

Mrs. Rosa, 92, with a mild NCD, loved flowers, and enjoyed going to the garden and naming the flowers. When realizing that I did not have time to go with her to the garden, she said “I know how to be patient, I’ll wait”. Mrs. Maria José saw me carrying a couple of folders and a laptop (material for the neuropsychological rehabilitation sessions), I approached her and she said to me: “go to your life, go to your pupils” (the neuropsychological rehabilitation sessions were designated as classes by some residents and the participants in the sessions as pupils).

Having time is also required due to a specificity of aging: The speed of information processing decreases (Firmino et al., 2016). With aging, reaction time increases, and the ability to use information stored in short-term memory decreases. These two conditions were observed during neuro-psychological rehabilitation sessions, and the residents themselves recognized they needed more time to complete the exercises. Mrs. Flôr, 75, with mild NCD, in a wheel-chair, recurrently made the following request: “don’t tell me, I’ll get it”. The same kind of demand was made by Mrs. Maria José: “I’m thinking, don’t tell me”.

Regrettably, lack of time is common in nursing homes, and is being identified as a difficulty in several countries (Kirtley & Williamson, 2016). Staff report they only have time to concentrate on basic needs (hygiene, nutrition, medication), leaving emotional and psychological needs, which require more time, unattended. The issue is that attending to those needs is essential for the well-being of every individual, including those living with dementia.

### Communication skills

Listening and interacting with older people living with dementia is a process that requires a greater awareness of how we listen and how we talk, and also knowledge of certain features of dementia (e.g., Eggenberger et al., 2013; Feil, 1992). This may be illustrated by four kinds of communication skills that can be helpful here.

First, one needs to pay more attention to the person’s idiolect, that is, to their particular way of speaking, especially the words that are used and the meanings attributed to the words. When we start to get acquainted with the person’s idiolect, we also start to get to know that person. We would also be able to interact using the same way of speaking. In this way we demonstrate we had paid attention, and we strengthen the relationship. Finally, interaction itself runs more smoothly and cheerfully when we speak in a similar manner.

These effects are present in all interactions. The argument here is they are valuable resources to rely upon in a context of cognitive impairments. Here are a couple of examples. Along a walk in the garden, I asked Mrs. Maria José the name of a plant, and she replied: “I identify it as a bougain-villea”. Bougainvilleas are not known by the majority of older Portuguese people and the vocabulary and syntax used belong to a formal register – all this showed her education and her focused attitude towards the question. Acknowledging this further guided my interaction with her.

Mrs. Genoveva, 96 years old, with a major NCD, called me and said: “yesterday I walked around the corridors as a dandy lady”. From then on I used the expression “dandy lady” to ask her about her walks. My role and name were not remembered, I was the person who used the same words, and this had the effect of establishing a bond between us.

Secondly, older adults living with dementia might not be able to explain historical and sociocultural references that are unknown for younger generations, and it is therefore useful to enlarge our culture knowledge to be able to understand and reply accordingly. Knowledge about the Portuguese dictatorship (1926-1974), the former Portuguese colonies, the role of women in the past, past famous politicians, or the names of household utensils no longer used represent a few examples. An excerpt of Mrs. Manuela’s discourse illustrates this: “I quite liked that thinner politician, the one who had another lady, they both died, I didn’t mind about her, she was beautiful. My husband was not like him, I suffered a lot, Ritinha^3^3Ritinha is an affectionate name for Rita., he was a whoremonger”. It helped to have the knowledge that Mrs. Manuela was talking about Francisco Sá Carneiro, the Portuguese prime-minister who had an extramarital relationship with Snu Abecassis, both dying in an aircraft crash in 1980.

Thirdly, one needs to be more aware and more sensitive to the interpretation and use of non-verbal communication. Several residents of the nursing home made use of manual wheelchairs, which they were unable to move. Whenever they wanted to talk to me, they waved or smiled at me, it was their way of saying “come here”. They were not able to get near me or call me by name, as some of them kept forgetting it, so I was simply called “Miss”. I got near, said “hi” with high intonation, smiled, maintained eye contact, and quite often gently touched the person’s head or hand. When verbal communication capacity is affected, it is even more important to learn to interpret non-verbal styles of communicating by older people living with dementia, and to encourage them to use this type of communication (Sabat, 2019).

Finally, a fourth communication skill is required by the fact that people living with dementia might experience different realities and beliefs that challenge our reality. Delusions is the technical term. Believing that the deceased father, mother, husband or wife are still alive, and asking to go home when there is no home to go to are common examples. A study by Kirtley and Williamson (2016) has brought important and useful information regarding the meaning of those experiences for the person themselves, and how caregivers should respond.

To get to the meaning of those experiences, one needs to understand that those experiences derive from three common themes: The person is using memory to make sense of the situation they are in; the person is expressing unmet needs, both physical and psychological; and the person is using creative or coping strategies (Kirtley & Williamson, 2016).

Mrs. Manuela, who had been in the hospital before entering the nursing home, used to say “I’m here to convalesce, I was very ill, I was brought here during the night, people are nicer here” – she was using her memory to make sense of the situation in which she found herself. Mr. António, 86 years old, with a major NCD, used to talk about his job as a cook as still existing and asked people to go and eat at his restaurant – he was communicating his need to be useful and active. Mrs. Maria do Céu, also 86 years old and with a major NCD, who had neuropsychological rehabilitation sessions on Tuesdays and Thursdays, refused a few times to go to the sessions, saying: “for me it’s not Thursday, I’m not going” – she was using a creative strategy to justify her refusal and avoid any pressure.

Besides identifying the themes behind those experiences of different realities and beliefs, caregivers also need to learn how to reply. Kirtley and Williamson (2016) portray five ways: telling the truth, looking for alternative meaning, distracting, going along with, and lying – this last possibility was recommended to be avoided whenever possible.

Understanding the themes and replying are interwoven in the interaction. Three examples follow. Mrs. Maria José’s husband was deceased but she used to ask when he would visit her. I replied by asking information about her husband, their wedding, and children. In doing so, I was looking for alternative meaning. At the same time, Mrs. Maria José’s unmet need for companionship and affection was being addressed.

Mr. António used to talk about his mother as if she were still alive. When I asked him about a special dish he still remembered, he became upset and reacted using the present tense “she makes this dish”. The way the interaction went on was by going along with, and attending to his unmet need of safety.

Mrs. Maria do Céu insisted she wanted to go home because her parents were waiting for her. I replied by distracting, proposing a walk in the garden, and attending to her unmet need of being cared for.

## Relatedness, Competence, and Autonomy

When we start listening to older people living with dementia, we see the needs of relatedness, competence, and autonomy, as conceptualized in self-determination theory ([SDT] Ryan & Deci, 2000). According to SDT, those needs are innate, and the development and well-being of human beings depend on their satisfaction. The need for relatedness leads us to connect, to establish bonds characterized by trust, safety, and intimacy. The need for competence is related to the experience of mastery and control upon the environment, it is boosted by positive feedback. The need for autonomy involves the determination to act in line with our self, values, principles and aspirations, and taking responsibility for our actions. All human beings actively seek to satisfy these three basic psychological needs. However, for that satisfaction to be achieved, social support is required. If there is no social support, needs will not be met, which has negative consequences for development and well-being.

Studies have been conducted to find out what happens with these needs in old age. Quantitative studies have shown that older people continue to experience them, and that their subjective well-being is dependent on the satisfaction of those needs: Positive correlations were found between ful-fillment of the three needs and subjective well-being (e.g., Neubauer et al., 2017; Firmino et al., 2016). Nonetheless, some differences were also observed in old age, in the hierarchy of importance of the three needs to subjective well-being: Relatedness came in the first place, followed by competence and finally autonomy (Neubauer et al., 2017). The relatively less important role of autonomy in older people’s well-being could be explained by the successful adaptation of older adults to an effective loss of autonomy, thus well-being is not as dependent on the satisfaction of that need (Neubauer et al., 2017).

What happens to these three needs in older adults living with dementia? My experience is that we when we listen to older people living with dementia we see the needs of relatedness, competence, and autonomy, and that subjective well-being is dependent on their satisfaction. This is supported by research (Beck et al., 2014; Deutscher Ethikrat, 2013; McCallion & Ferretti, 2017). Research also stresses the importance of the satisfaction of those needs to minimize the consequences of the NCD in the person’s life. Relatedness, specially, is pointed out as a need that continues to be experienced in older adults living with dementia, being even as imperative as in childhood (Kitwood, 1997/2019b). Dementia can cause disorientation and confusion, states that activate the attachment behavioral system (Cheston, 2019).

Living with dementia decreases the opportunity for satisfying the three needs considerably. People lose various cognitive abilities, becoming more and more dependent. Thus, the possibility of satisfaction depends largely on how care is provided, and what the institutional approach to care is like (Beck et al., 2014; Deutscher Ethikrat, 2013; Grabowski et al., 2014). Care should be provided based on the assumption that older adults living with dementia continue to experience the three basic psychological needs, and adjusted to help fulfill those needs. The institutional approach to care should be personcentered.

### Relatedness

How does a psychologist working in a nursing home help residents fulfill the need for relatedness? It is through a therapeutic relationship which is different from the therapeutic relationship built in other therapeutic contexts. The therapeutic relationship with older adults living with dementia is characterized by emotional support and human warmth (Firmino et al., 2006). Its main guiding principles are validation and reassurance, along with providing practical help to accomplish certain tasks (Cheston, 2019; Feil, 1992; Kitwood, 1997/2019c).

Let us listen. Mrs. Flôr, after a long weekend, greeted me by saying: “I’ve missed you”. Mrs. Eva, 76 years old, with a mild NCD: “I miss you when you are not around. If I were to decide you would always be near me”. Mrs. Maria José: “with you I don’t feel lonely”. Mrs. Manuela: “I don’t know how to thank you for this affection and friendship”; “I know that this girl appreciates me, I can see that”.

Studies show that what older adults living with dementia value the most is an affectionate and compassionate way of interacting (Kirtley & Williamson, 2016), which is also illustrated by these voices. Studies also demonstrate how older adults living with dementia are able to make new friends and build long-term memories of those who treat them with affection, even if they do not remember the person’s name or role (Sabat, 2019).

The nature of this therapeutic relationship carries a deontological dilemma: the dilemma between the principle of *Beneficence and non-maleficence* and the principle of *Responsibility* (Ordem dos Psicólogos Portugueses – Portuguese Psychologists Association, n.d.). A caring and affectionate therapeutic relationship has a positive value for older people living with dementia, and it could cause them harm if one expressed unwillingness to comply with such a relationship (principle of Beneficence and non-maleficence). At the same time, negative consequences might arise as we are dealing with very vulnerable people who can become too dependent on us (principle of Responsibility). An attempt to solve the dilemma is by finding a balance between giving affection and promoting autonomy. Another way is to promote more closeness between residents and their family and friends, as well as with staff.

Human beings build relationships through interaction, stories they tell each other, and also through touch – it is the same with older people living with dementia.

#### Interaction

A basic way of relating to others is by talking. Nursing homes frequently provide care to several people, who are together in various areas of the house. People are also involved in group activities. All this could suggest there is a lot of talking, but in reality there is not. Few residents in nursing homes are capable of initiating and maintaining a dialogue, least building a relationship. Thus, the need for relatedness is difficult to fulfill through interaction. How can one help?

First, it is necessary to understand that human beings are semiotic beings, which means that we (i) have the capacity to express ourselves verbally and non-verbally; (ii) act intentionally when we interact with others; and (iii) seek to cooperate with our interlocutors. Secondly, it is necessary to realize that semiotic behavior does not vanish when living with dementia (Deutscher Ethikrat, 2013; Sabat, 2019; Sabat & Cagigas, 1997). Our attitudes and behaviors when inter-acting with older people living with dementia should pre-suppose these two guidelines. The already mentioned communication skills should also be present. Guided by all this, our interaction helps to satisfy the need for relatedness. Let us listen.

Mrs. Manuela: “one day you’ll get sick and tired of listening to us”; “only with Ritinha I feel comfortable saying these things”. Mrs. Maria José: “I only confide in you”. One day, when I was leaving work and Mrs. Maria José was getting ready for supper, she asked me: “have you got supper at home?” Another day she made a comment on a skirt I was wearing: “I like your skirt. Where did you buy it? Was in a sale?”, and we went on talking about shopping and sales. Another day I complimented her on her hands, saying she had the hands of a pianist, to which she replied laughing: “I play the piano and I speak French”^4^4This is a Portuguese phrase used in the past to refer to the requirements of an educated woman.

As with other people, interaction with older adults living with dementia is not free of conflict and negative feelings. Mrs. Maria, 85 years old, with a major NCD, seeing me talking to Mrs. Manuela, called and complained: “you only care about that one there, she is luxury and I’m garbage”. It took time to change this belief and the experience of feeling left out.

It is also important to try to promote interaction among residents, bearing in mind that help from us is required. Two examples follow.

Mrs. Manuela and Mrs. Maria José were face to face in their wheelchairs, I was in the middle. Mrs. Manuela was saying that she used to make a type of cheese, starting to explain how it was done, but without mentioning its name, and at some point Mrs. Maria José interrupted and said “cream cheese”.

Mrs. Maria do Céu and Mrs. Flôr were face to face in their wheelchairs, I was in the middle. We were talking and, at one moment, all three looked outside. Mrs. Flôr commented it was a beautiful day and that she would like to go outside. Mrs. Maria do Céu replied “you can take her, I don’t mind, I like her”.

#### Stories

In dementia, short-term memory is much more affected than semantic, autobiographical, and implicit memory (APA, 2013), which allows older adults living with dementia to remember facts, events, and feelings from the past.

When a story is told, the action of narrating is set in a now-moment that is created and shared by the one who tells the story and the one who listens to it, bringing the possibility of building a connection between the two (Estrada, 2014). I have listened to stories about weddings, children, work, parents, siblings, significant events, school, recipes.

Let us listen again. Mrs. Manuela: “I had a happy marriage, it was happy on my own way. Living, having a life, I don’t even know how I’ve lived. I speak honestly now: My daughter was born because she was due to be born. Life is not a bed of roses”. Mrs. Rosa: “my dream was to be independent after losing my dad, mom, and sister who was like a mother to me, but then I got married…”

Research about reminiscence therapy, which involves

recalling past events and is used with older people living with dementia, has shown that moments of storytelling between caregivers and older people living with dementia made caregivers become connected with those people, and start to see the person through the dementia (Cooney et al., 2014).

#### Touch

Touching and being touched along with social interaction bring positive effects to physical and psychological well-being, and to the interpersonal relationship itself (Field, 2010; Jakubiak & Feeney, 2016). At the physiological level, effects occur in the decrease of blood pressure and heart rate, and at the biochemical level cortisol decreases and oxytocin increases (Field, 2010). Touch promotes psychological well-being, in the form of positive mood and subjective well-being (Suvilehto et al., 2019). Additionally, touch contributes to interpersonal well-being, because it is perceived as an indicator of relational connection, affection, and care (Suvilehto et al., 2019). Who, how, and when we touch, and on which parts of the body, depend largely on the type of the relationship, social situation, and culture (Field, 2010; Suvilehto et al., 2019), and of course not all individuals want to be touched or find it comforting.

Touch is also present in the care of older adults in nursing homes, provided by nurses (Routasalo & Isola, 1996) and care assistants (Mononen, 2019). Research has shown that older people experience touch as comforting and caring; interaction between caregiver and older adults becomes smoother, and both parties feel more motivated to build a positive relationship (Mononen, 2019; Routasalo & Isola, 1996).

As a psychologist in a nursing home using validation therapy and person-centered care for people living with dementia, it is easily noticeable how touch is important for residents (see also Kitwood, 1997/2019c). It is important to satisfy the need for relatedness, to provide all the benefits that touch brings to human beings, and to foster the therapeutic relationship. Touch is also useful to gain and sustain attention, and to calm and reassure (Feil, 1992; Lévy-Storms, 2012; Mononen, 2019).

Stroking, putting my hand on a person’s shoulder or arm, offering my arm to walk, and giving my hand are different ways of touching female adults living with dementia (most of the older adults were women). With male adults, putting my hand on the shoulder or arm was culturally allowed and accepted with contentment. The effects of touch were noticed in the therapeutic relationship in the form of more connection, more trust, more openness (Phelan, 2009).

Touching and non-verbal communication may be the only way to communicate for some residents. This was the case with Mrs. Conceição, 82 years old, who had major NCD and a major speech function disturbance. On one occasion I felt her hand on my back. When I turned to her, she asked me with gestures to turn back again. I did that, and I felt her adjusting my sweater with her hands. I turned again to her and thanked her, and she smiled. It was a rare moment of communication between us.

During the pandemic, the use of touch has become very difficult, if not impossible, in nursing homes. The use of protective suits and masks constitutes an extreme limitation on its benefits.

### Competence

The need for competence is noticed particularly when older people living with dementia are performing a task, for instance during the neuropsychological rehabilitation sessions. The wish to get it right is visible. Residents use to ask if they are “doing well”, and they enjoy showing that they know an answer. Let us listen.

Mrs. Laura, 91 years old, with a major NCD, was constantly commenting: “I got it right, I did everything well”. Along a task of identifying animals, she started singing a Portuguese song after seeing a seagull, and she said with pride “I sing well, don’t I?”

Residents also realize that sometimes they do not know the answer to the tasks. Interestingly, they reveal coping strategies in dealing with those situations where they lack competence in performing a particular task. During a task of identifying objects Mrs. Manuela suggested “you could write down the name of each thing, so I wouldn’t make any mistakes”. In a time orientation task she said “I want to ask you a favor, I want you to get me a calendar”. In both situations Mrs. Manuela used problem-focused coping. Once again in a time orientation task, Mrs. Célia, 92 years old, with a major NCD, after hearing that the year we were in was 2020, said laughing: “2020? Already? We are late!” Here humor was used.

During a memory task Mr. Aníbal, 80 years old, with a mild NCD, proposed: “I’m going to do this to you now, see if you get it right”. I accepted changing roles and I perceived this taking over as an expression of competence. On another occasion Mr. Aníbal said: “girl, you know I like to be active, to keep my mind active, but I can hardly hear you, I need you to speak up”.

A simple and daily task most women residents wished to perform was tidying up their clothes. They made it clear they were competent in that, and they wanted to do things their way. Sometimes help was given, but just the necessary amount.

Older people living with dementia also show their need for competence during interaction. Let us listen to Mrs. Manuela. One day we were talking about flowers, and how pretty some flowers were. Then Mrs. Manuela announced proudly: “I know a fruit that is not a flower but it is as pretty as flower: cherries!” At another time, when she was watching some news about Isabel dos Santos^5^5Isabel dos Santos is the daughter of the former President of Angola, but as it happens her surname is fairly common. and the Luanda Leaks, she called me and commented: “Isabel dos Santos is not a celebrity name”. When the pandemic spread and visits to the nursing home ceased to be allowed, I explained this to Mrs. Manuela, who replied “I understand things much more complicated than that”. All these extraordinary remarks were made by a 98-year-old woman with severe dementia, and they revealed her need for competence and her actual competence.

The acknowledgment of the progressive loss of competencies also emerged in some cases, and it was hurtful. It was the case of Mrs. Maria José, who started to be fed as she was not able to do it herself due to the severity of her motor disturbance: “I’m very dependent, I feel ashamed”. Older adults living with dementia also used to compare what they were able to do in the past to what they were able to do in the present, regretting the losses, thereby showing their need for competence.

It is important to note that satisfying the need for competence of older adults living with dementia can bring risks for them, creating a dilemma between satisfying the need for competence and ensuring security. It is recommended to strike a balance between the two sides (American Bar Association Commission on Law and Aging & American Psychological Association, 2008).

### Autonomy

The need for autonomy is clearly expressed by the words of the American psychologist Richard Taylor, with Alzheimer’s disease: “what about my need and desire to maintain some of my own independence and feel and act like I am an almost completely functioning adult” (2007, p. 212). At the same time, research findings with older adults have shown that the need for autonomy is not correlated with subjective well-being, which is explained by the successful adaptation of older adults to the effective loss of their autonomy (Neubauer et al., 2017). From my experience in the nursing home, I observed residents for whom loss of autonomy was accepted without suffering or embarrassment and residents who, like Richard Taylor, pursued to satisfy their need for autonomy.

The need for autonomy was often expressed in decision making. Residents expressed their will about routine decisions, and wished it to be respected: going for a walk, having a midmorning snack, drinking water, dyeing their hair, staying in bed, taking a nap, watching TV.

Additionally, some residents expressed their need for autonomy in more elaborated ways. Let us listen. Mrs. Maria José: “I’m pondering here, today is Wednesday, it’s market day, let’s go there”. Mrs. Maria José used the verb “to ponder” that expresses intentionality, made an invitation, and, although that day was not Wednesday, market day was in fact on Wednesday. Mr. Aníbal, who had a swallowing disorder, expressed his willingness to cease eating soft diet. He was aware of the risks associated with that decision: “I’ll have it written down I want to eat a normal diet and that I’m aware I can die. I’ll have it written down. I’ll bear the consequences”.

The need for autonomy was also expressed in the desire to go home, even if there was no home to go to. It was a desire filled with suffering. Mrs. Manuela: “I’m also right, Ritinha [I had said her son was right in observing that she could not be alone at home], I want to go home, I want my things”. Mrs. Maria José: “I want you, lass, to get me out of here”.

In order to satisfy the need for autonomy of older adults living with dementia, as it happens with the need for competence, security might be put in danger. There is a dilemma here and it must be acknowledged. Incapacity to identify this dilemma and embracing security jeopardizes a minimum of satisfaction of the need for autonomy or competence and thus well-being. That is common under standard conditions, having become worse during the pandemic. The dilemma is rarely identified, hindering a debate about the negative effects of security measures in nursing homes.

## Conclusion

When we care for older adults living with dementia, we run the risk of focusing only on the diagnosis itself – dementia. But has the person vanished? Has their identity, life story, likes and dislikes been erased? The answer is no. The person is there, as the voices of all the adults living with dementia quoted in this paper have demonstrated. We just have to listen and follow the framework of validation therapy and person-centered care for people living with dementia. The process of listening is not simple or easy: Time and communication skills are required.

As the person is there, so are the needs for relatedness, competence, and autonomy, and satisfaction of those needs is essential for well-being. As dementia brings dependence, caregivers are called to help in obtaining that satisfaction. As a psychologist in a nursing home, I have faced dilemmas in helping the residents in satisfying their needs: satisfying the need for relatedness vs. aggravating dependence; satisfying the need for competence and autonomy vs. assuring security.

Two conclusions come to surface. First, seeing the person through the dementia allows us to obtain an adequate psychological assessment, and to delineate a helpful supportive psychotherapy. Diagnosis itself and neuropsychological tests are not enough; listening and interacting with the person in their natural social environment is essential. Along this process, a caring attitude is a must. Secondly, seeing the person through the dementia also makes us acknowledge and help satisfy the basic psychological needs for relatedness, competence, and autonomy. Until we die, we are a person.

## Action editor

Lars-Gunnar Lundh served as action editor for this article.
